# Investigating genotype by environment interaction for beef cattle fertility traits in commercial herds in northern Australia with multi-trait analysis

**DOI:** 10.1186/s12711-024-00936-0

**Published:** 2024-10-31

**Authors:** James P. Copley, Benjamin J. Hayes, Elizabeth M. Ross, Shannon Speight, Geoffry Fordyce, Benjamin J. Wood, Bailey N. Engle

**Affiliations:** 1 Centre for Animal Science, Queensland Alliance for Agriculture and Food Innovation, St Lucia, QLD 4072 Australia; 2 Black Box Co, Mareeba, QLD 4880 Australia; 3 School of Veterinary Science, University of Queensland, Gatton, QLD 4343 Australia; 4 USDA, ARS, U.S. Meat Animal Research Center, Clay Center, NE 68933 USA

## Abstract

**Background:**

Genotype by environment interactions (GxE) affect a range of production traits in beef cattle. Quantifying the effect of GxE in commercial and multi-breed herds is challenging due to unknown genetic linkage between animals across environment levels. The primary aim of this study was to use multi-trait models to investigate GxE for three heifer fertility traits, *corpus luteum* (CL) presence, first pregnancy and second pregnancy, in a large tropical beef multibreed dataset (n = 21,037). Environmental levels were defined by two different descriptors, burden of heat load (temperature humidity index, THI) and nutritional availability (based on mean average daily gain for the herd, ADWG). To separate the effects of genetic linkage and real GxE across the environments, 1000 replicates of a simulated phenotype were generated by simulating QTL effects with no GxE onto real marker genotypes from the population, to determine the genetic correlations that could be expected across environments due to the existing genetic linkage only. Correlations from the real phenotypes were then compared to the empirical distribution under the null hypothesis from the simulated data. By adopting this approach, this study attempted to establish if low genetic correlations between environmental levels were due to GxE or insufficient genetic linkage between animals in each environmental level.

**Results:**

The correlations (being less than <0.8) for the real phenotypes were indicative of GxE for CL presence between ADWG environmental levels and in pregnancy traits. However, none of the correlations for CL presence or first pregnancy between ADWG levels were below the 5th percentile value for the empirical distribution under the null hypothesis from the simulated data. Only one statistically significant (*P* < 0.05) indication of GxE for first pregnancy was found between THI environmental levels, where r_g_ = 0.28 and 5th percentile value = 0.29, and this result was marginal.

**Conclusions:**

Only one case of statistically significant GxE for fertility traits was detected for first pregnancy between THI environmental levels 2 and 3. Other initial indications of GxE that were observed from the real phenotypes did not prove significant when compared to an empirical null distribution from simulated phenotypes. The lack of compelling evidence of GxE indicates that direct selection for fertility traits can be made accurately, using a single evaluation, regardless of environment.

**Supplementary Information:**

The online version contains supplementary material available at 10.1186/s12711-024-00936-0.

## Background

Beef production in tropical pastoral systems, such as northern Australia, takes place in environments ranging from hot, dry and arid to those with very high rainfall and humidity. Given the diversity of environments in tropical pastoral systems, there is value investigating whether genotype by environment interaction (GxE) could be considered in genetic evaluations to improve the accuracy of selection in a target environment. In the presence of GxE, genotypes will respond to environmental variation differently [1–3]. GxE is important for all breeding objectives but particularly for fertility traits, as these traits are key drivers of profitability in tropical beef enterprises and are affected by heat and nutritional stress [4, 5]. If ignored it may decrease the accuracy of estimated breeding values, but when accounted for, GxE can be used positively as it provides a source of genetic variation [6, 7]. This can affect targeted selection in two ways: re-ranking and re-scaling of breeding values across environments [2, 6, 8]. Re-scaling of breeding values from one environment to another means that while the estimates of genetic merit of individual animals may change, the overall ranking of animals is not affected [1, 3, 9]. Re-ranking occurs when the relative genetic merit of individuals changes from one environment to the other [3, 6]. The latter creates a problem for livestock production systems where stud breeders are supplying the commercial sectors. If stud bulls, for example, are selected under a vastly different environment compared to the commercial environment where they will be used, the progeny may be sub-optimal for that commercial environment. This would be particularly important where animals are spread over large geographical regions with very different environments.

A common method to estimate GxE is to consider the same trait measured in different environments as two different traits [3, 6, 10]. In this multi-trait approach, a low correlation (<0.8), between the same trait measured in two separate environments is indicative of GxE [3, 9]. Multi-trait models have been used successfully to examine GxE interaction in numerous studies [2, 10–16]. If the genetic correlation is <0.8, then it is beneficial to a breeding program to actually split selection according to environments, resulting in a faster rate of genetic gain [17]. However, GxE is often not considered when calculating breeding values, as it is challenging to detect and measure outside of carefully designed studies that often balance the progeny of sires across different environments [2, 6, 18]. GxE will often be estimated from data collected in small, intensively recorded herds where the inter-herd genetic linkage is known and understood from the pedigree [2, 8, 14, 19]. Sufficient linkage, where the same or closely related genotype is present in multiple environments, is critical to successfully conduct GxE investigations [20]. Calculating the genetic correlation between environments with insufficient linkage will result in lower correlations (<0.8) and this can potentially overestimate the true GxE [3]. The lower genetic correlation between environments in the multivariate approach can arise either from inadequate genetic linkage between environments, actual GxE between environments, or a combination of both.

The absence of reliable estimates for the magnitude of linkage between herds has limited the utility of the multi-trait approach when applied to commercial beef datasets. In extensive Australian pastoral systems, it is quite normal for parentage or pedigree to be entirely unknown [5]. Using pedigree information to estimate GxE relies on the progeny of multiple sires being represented across the different environments. Using genomic information to estimate GxE is potentially a solution for situations where pedigree is unavailable and may have more power to detect GxE [7]. This is because the requirement in a genomic analysis is for chromosome segments or single nucleotide polymorphism (SNP) alleles to be replicated across environments, as opposed to progeny in a pedigree analysis [3].

The primary objective of this study was to investigate the presence of GxE interactions in a large, unstructured, industry-derived, beef cattle dataset for female fertility. We hypothesized that, given the significant environmental challenges across a range of environments in the data set, GxE interaction between female fertility and both feed availability and/or prolonged heat exposure would be observed. We took a multi-trait approach to assessing the potential for GxE. To disentangle the effects of genetic linkage from true GxE on the estimates of genetic correlations between environments, we simulated 1000 replicates of a quantitative trait under the null hypothesis of no GxE between the environments, using the true genotypes of the test population to capture the genetic linkage between environments. Estimates for GxE, as measured by genetic correlation from the real phenotypes and genotypes, were then compared to an empirical distribution of correlation estimates from the simulated data. Correlation estimates from the real phenotypes that fell within the 5th percentile of the distribution were interpreted as statistically significantly different from unity (*P* < 0.05).

## Methods

### Phenotypes and project collaborators

**Fig. 1:**
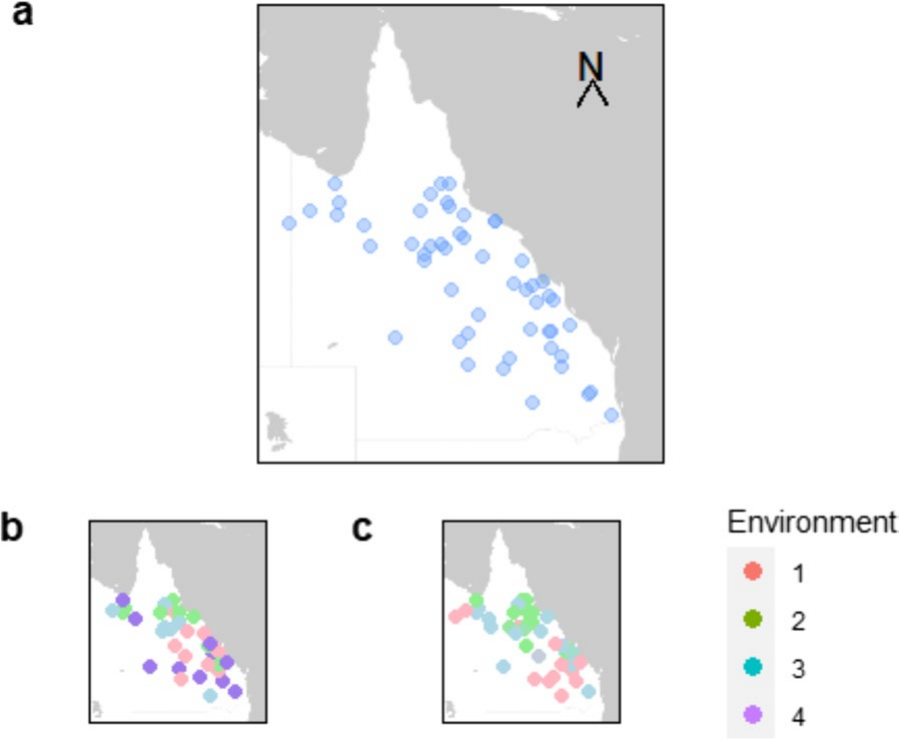
Project collaborators and environmental levels. **a** Map showing the locations of the 54 collaborating herds; **b** Locations colour-coded according to Average Daily Weight Gain environmental levels, 1 = harshest, 4 = most favourable; **c** Locations colour-coded according to Temperature Humidity Index environmental levels, 1 = harshest, 3 = most favourable

The phenotypes and genotypes used in this study were collected as part of the Northern Beef Genomics project [4, 15, 21]. This project collected 21,037 paired phenotypes and genotypes from animals managed under diverse conditions from 54 commercial beef businesses representative of northern Australia (Fig. 1a).

Three fertility phenotypes, *corpus luteum* presence (CL presence), first pregnancy, and second pregnancy were recorded over a 3-year period. CL presence, the first trait recorded, was an ovarian ultrasound procedure conducted at approximately 600 days of age, a binary measure where; 1 = “pubertal” and 0 = “non-pubertal” [21, 22]. First and second pregnancy were measured at approximately 2.5 years and 3.5 years of age, respectively. Testing was conducted via manual palpation or ultrasound examination by veterinarians and project collaborators at a time post-bull exposure that fit with the collaborator’s typical management schedule. Pregnancy was also modelled as a binary trait (1 = “pregnant”, 0 = “non-pregnant”) [4]. Throughout the analysis, these binary traits were examined as continuous responses using mixed linear models, as has been extensively utilized in the literature [23–26]. Previous efforts to fit threshold and more complicated models to this dataset [4] revealed the advantages of the linear model approach for convergence and interpretability, particularly in a multivariate model. Given the distribution of cases and controls is nearly balanced for each trait and the large sample size, a normal distribution is expected to approximate the binary trait. Finally, this approach provided continuity with other examinations of the dataset [4, 15].

In conjunction with fertility trait recording, additional covariates were recorded for each individual animal:

#### Weight

Animal liveweight (body weight) on the day of each fertility trait recording at CL presence, first pregnancy and second pregnancy.

#### Hip height

Distance from the ground to the peak of the sacrum [4]. Hip Height was measured at the time of each fertility recording.

#### Body condition score (BCS)

Defined on a 1–5 scale with 1 being poor condition to 5 being fat. BCS was evaluated at the time of each fertility trait recording.

These three covariates were fit in the multi-trait analyses. These covariates have previously been tested for significance to the traits by [4].

### Environmental descriptors

The environmental descriptors were based on nutritional availability, represented here as average daily weight gain (ADWG) of the herd, and cumulative heat load experienced beyond a defined temperature-humidity index (THI) threshold of 65 [4, 16, 27]:

$$\text{THI}=0.8*\text{T}+\left(\left(\text{RH}*0.01\right)*\left(\text{T}-14.4\right)\right)+46.4.$$

ADWG was calculated for each individual animal based on the liveweight records from consecutive fertility measurements, CL presence to first pregnancy (typically 6 to 12 months and usually including one annual growing season) and first pregnancy to second pregnancy (typically 1 year) [4]. A limitation of this environmental descriptor was inconsistency of the timing between trait recordings, however, the lack of more precise nutritional information or additional liveweight records meant that adopting ADWG was the only viable nutritional descriptor in this complex industry dataset. The ADWG of each individual animal (kg/day), from CL presence to first pregnancy and from first pregnancy to second pregnancy, was used to calculate the phenotypic mean ADWG of each contemporary group. Contemporary groups were defined as combinations of farm/collaborator and year. Based on the mean ADWG value, each contemporary group was then assigned into quartiles from 1 (lowest ADWG) to 4 (highest ADWG).

Another limitation of this method was that no liveweight records were made before CL presence recording. While liveweight was always recorded at CL presence, it was not recorded prior to this because CL presence recording was the first time that the project had access to the animals. Consequently, ADWG could not be calculated for the time-period preceding CL presence recording and environmental levels for this trait could not be defined directly. As an alternative, the CL presence records were ‘binned’, by contemporary group, into the same environmental levels as for first pregnancy. This effectively assigned animals to a quartile representing their ‘future’ performance. The use of future ADWG performance, while lacking precision, was still indicative of nutritional availability for the respective herd. It must be noted that contemporary groups of animals only changed ADWG environmental levels seven times, from a total of 80 contemporary groups, from first to second pregnancy. These seven contemporary groups represented a total of 497 animals. Thus, whilst the use of future ADWG performance to run a GxE analysis for CL presence was not perfect, it was the best solution available within the industry dataset. The ADWG levels for second pregnancy were defined from the weight gain from first to second pregnancy.

Days over a THI threshold was defined on an individual animal basis, representing chronic heat load during critical reproductive timepoints. The descriptor was the number of days where the THI threshold of 65 was exceeded within the 120-day period surrounding (60 days prior and 60 days post) (1) trait recording for CL presence, or (2) conception dates, in the case of both first and second pregnancies [4]. Despite the low threshold, this THI was selected as a proxy for relative environmental harshness. The temperature and humidity data for THI calculation [4] was obtained with the NASAPOWER package in R v3.5.4; the data was interpolated from direct weather observations to a 0.5 by 0.5 degree grid of latitude and longitude [28]. The full explanation of THI as an environmental descriptor is available from [4]. The same procedure described for allocating ADWG quartiles was followed here to disperse animals into terciles with 1 (longest exposure to heat load) to 3 (shortest exposure to heat load) based on the mean within each contemporary group. Three rather than four levels were selected for characterizing THI due to insufficient environmental differentiation between quartile levels.

### Genotypes

All animals were genotyped on 35K GGP tropBeef SNP array (Neogen Australasia, Ipswich, QLD), which was specifically developed for use on tropical and tropical composite breeds to reduce ascertainment bias. Genotypes with GC score less than 0.6 were set to missing. Genotypes were imputed up to 709,768 SNPs (Bovine HD array) using the FImpute software [29] and a panel of 3,140 cattle of relevant breeds genotyped for the Bovine HD array [15]. A principal component analysis (PCA) was conducted using GCTA, six principal components were calculated and then plotted using ggplot2 in R 4.2.2 [30, 31]. Estimates of genetic distance, F_st_, were made as pairwise comparisons between sub-populations, in this case the different environmental levels. These were estimated in GCTA [30, 32]. The F_st_ analysis was conducted as a preliminary examination of genetic linkage between environmental levels.

### Genomic covariates

Breed composition, heterozygosity, and *Bos indicus* content were calculated from the genotypes. Breed proportion aimed to capture the genetic composition of each animal in relation to 14 breeds common in northern Australia and represented in the dataset: Angus, Belmont Red, Brahman, Charolais, Droughtmaster, Hereford, Limousin, Murray Gray, Santa Gertrudis, Shorthorn, Wagyu, Boran, Senepol and Tuli. Individual breed proportions were estimated using a separate data set comprised of purebred cattle from the 14 breeds [15]. SNP effects for breed composition were estimated using a genomic best linear unbiased prediction model, where the phenotype was 1 if the animal was of that breed and 0 if not [15, 33]. The effects of each SNP for the proportion of each breed were then derived by back-solving for the SNP effects [30], and the resulting prediction equations for each breed were used to estimate breed proportions in the dataset [15]. The breed composition for each of 14 breeds was then included in the model. *B. indicus* percentage, or proportion of the genome estimated to be of *B. indicus* origin, was calculated using the same method as breed composition, the only difference being the use of a single, pure *B. indicus* reference dataset instead of one comprising of individual breeds [15]. Marker heterozygosity for each animal was fitted as a covariate to account for heterosis [15] and ranged from 0.25 (purebred) to 0.5 (F_1_ cross) [4, 15, 21]. Breed proportions, *B. indicus* content, and heterozygosity were previously calculated by Hayes et al. [15] for this dataset and were included in the multi-trait model as covariates.

### Heritability

Heritability was first estimated using a univariate model, ignoring environment, to establish the baseline heritability for each trait. Heritability was then re-estimated within each environmental level. Estimation in all cases was done using the model:

$$\mathbf{y}=\mathbf{X}{\varvec{\upbeta}}+\mathbf{Z}{\varvec{\upmu}}+\mathbf{e}.$$

In the equation, $\mathbf{y}$ = vector of phenotypes (CL presence, first pregnancy, or second pregnancy), $\mathbf{X}$ = design matrix allocating phenotypes to fixed effects, ${\varvec{\upbeta}}$ = vector of fixed effects, including weight, hip height, BCS, 14 breed composition vectors, heterozygosity, and percentage *B. indicus* as covariates and contemporary group; $\mathbf{Z}$ = design matrix allocating records to animal effects; $\mathbf{u}$= vector of random additive genetic animal effects, one for each animal, distributed $N(\mathbf{0},\mathbf{G}{\upsigma }_{\text{u}}^{2})$; and, $\mathbf{e}$ = vector of residual errors, distributed as $N(\mathbf{0},\mathbf{I}{\upsigma }_{\text{e}}^{2})$. The $\mathbf{G}$ matrix is the genomic relationships among the animals, calculated from the SNP data [30]. Univariate REML in MTG2 was used to estimate the variance components and the heritability for each trait [34].

As discussed by [60] **G**⦻**T** is not strictly a genetic covariance matrix (for reasons including that is the variance captured by SNP rather than the full additive genetic variance for this trait, and the fact that genotyped SNP may not have the same allele frequency distribution as casual variants), so that this approach is actually equivalent to a Bayesian prior belief (rather than a classic mixed model for BLUP).

### Genomic correlations

Assessing GxE was done by calculating the genomic correlation between the same trait in different environmental levels, with each environmental level treated as a separate trait [1, 35], using the model:

$$\left[\begin{array}{c}{\mathbf{y}}_{\mathbf{A}\mathbf{D}\mathbf{W}\mathbf{G}1}\\ {\mathbf{y}}_{\mathbf{A}\mathbf{D}\mathbf{W}\mathbf{G}2}\\ {\mathbf{y}}_{\mathbf{A}\mathbf{D}\mathbf{W}\mathbf{G}3}\\ {\mathbf{y}}_{\mathbf{A}\mathbf{D}\mathbf{W}\mathbf{G}4}\end{array}\right]= \left[\begin{array}{cccc}{\mathbf{X}}_{\mathbf{A}\mathbf{D}\mathbf{W}\mathbf{G}1}& & & \\ & {\mathbf{X}}_{\mathbf{A}\mathbf{D}\mathbf{W}\mathbf{G}2}& & \\ & & {\mathbf{X}}_{\mathbf{A}\mathbf{D}\mathbf{W}\mathbf{G}3}& \\ & & & {\mathbf{X}}_{\mathbf{A}\mathbf{D}\mathbf{W}\mathbf{G}4}\end{array}\right] \left[\begin{array}{c}{{\varvec{\upbeta}}}_{\mathbf{A}\mathbf{D}\mathbf{W}\mathbf{G}1}\\ {{\varvec{\upbeta}}}_{\mathbf{A}\mathbf{D}\mathbf{W}\mathbf{G}2}\\ {{\varvec{\upbeta}}}_{\mathbf{A}\mathbf{D}\mathbf{W}\mathbf{G}3}\\ {{\varvec{\upbeta}}}_{\mathbf{A}\mathbf{D}\mathbf{W}\mathbf{G}4}\end{array}\right]+\left[\begin{array}{cccc}{\mathbf{Z}}_{\mathbf{A}\mathbf{D}\mathbf{W}\mathbf{G}1}& & & \\ & {\mathbf{Z}}_{\mathbf{A}\mathbf{D}\mathbf{W}\mathbf{G}2}& & \\ & & {\mathbf{Z}}_{\mathbf{A}\mathbf{D}\mathbf{W}\mathbf{G}3}& \\ & & & {\mathbf{Z}}_{\mathbf{A}\mathbf{D}\mathbf{W}\mathbf{G}4}\end{array}\right] \left[\begin{array}{c}{\mathbf{u}}_{\mathbf{A}\mathbf{D}\mathbf{W}\mathbf{G}1}\\ {\mathbf{u}}_{\mathbf{A}\mathbf{D}\mathbf{W}\mathbf{G}2}\\ {\mathbf{u}}_{\mathbf{A}\mathbf{D}\mathbf{W}\mathbf{G}3}\\ {\mathbf{u}}_{\mathbf{A}\mathbf{D}\mathbf{W}\mathbf{G}4}\end{array}\right]+ \left[\begin{array}{c}{\mathbf{e}}_{\mathbf{A}\mathbf{D}\mathbf{W}\mathbf{G}1}\\ {\mathbf{e}}_{\mathbf{A}\mathbf{D}\mathbf{W}\mathbf{G}2}\\ {\mathbf{e}}_{\mathbf{A}\mathbf{D}\mathbf{W}\mathbf{G}3}\\ {\mathbf{e}}_{\mathbf{A}\mathbf{D}\mathbf{W}\mathbf{G}4}\end{array}\right],$$

where: ${\mathbf{y}}_{\text{ADWG}(1-4)}$ = vectors of fertility phenotypes within each environmental level, ${\mathbf{X}}_{\text{ADWG}(1-4)}$ = incidence matrices for the fixed effects, ${{\varvec{\upbeta}}}_{\text{ADWG}(1-4)}$**=** vector of fixed effects, ${\mathbf{Z}}_{\text{ADWG}(1-4)}$ = incidence matrices for the random additive genetic animal effect, ${\mathbf{u}}_{\text{ADWG}(1-4)}$ = vectors containing random additive genetic effects, ${\mathbf{e}}_{\text{ADWG}(1-4)}$ = vectors of random residual effects.

The random effects ${\text{u}}_{\text{ADWG}(1-4)}$ were distributed as $$\left[\begin{array}{c}{\mathbf{u}}_{\mathbf{A}\mathbf{D}\mathbf{W}\mathbf{G}1}\\ {\mathbf{u}}_{\mathbf{A}\mathbf{D}\mathbf{W}\mathbf{G}2}\\ {\mathbf{u}}_{\mathbf{A}\mathbf{D}\mathbf{W}\mathbf{G}3}\\ {\mathbf{u}}_{\mathbf{A}\mathbf{D}\mathbf{W}\mathbf{G}4}\end{array}\right]\sim N(0,\mathbf{G}  \otimes  \mathbf{T})$$, where $$\mathbf{T}=\left[\begin{array}{cccc}{\upsigma }_{{\text{u}}_{\text{ADWG}1}}^{2}& {\upsigma }_{{\text{u}}_{\text{ADWG}2},{\text{u}}_{\text{ADWG}1}}& {\upsigma }_{{\text{u}}_{\text{ADWG}3},{\text{u}}_{\text{ADWG}1}}& {\upsigma }_{{\text{u}}_{\text{ADWG}4},{\text{u}}_{\text{ADWG}1}}\\ {\upsigma }_{{\text{u}}_{\text{ADWG}1},{\text{u}}_{\text{ADWG}2}}& {\upsigma }_{{\text{u}}_{\text{ADWG}2}}^{2}& {\upsigma }_{{\text{u}}_{\text{ADWG}3},{\text{u}}_{\text{ADWG}2}}& {\upsigma }_{{\text{u}}_{\text{ADWG}4},{\text{u}}_{\text{ADWG}2}}\\ {\upsigma }_{{\text{u}}_{\text{ADWG}1},{\text{u}}_{\text{ADWG}3}}& {\upsigma }_{{\text{u}}_{\text{ADWG}2},{\text{u}}_{\text{ADWG}3}}& {\upsigma }_{{\text{u}}_{\text{ADWG}3}}^{2}& {\upsigma }_{{\text{u}}_{\text{ADWG}4},{\text{u}}_{\text{ADWG}3}}\\ {\upsigma }_{{\text{u}}_{\text{ADWG}1},{\text{u}}_{\text{ADWG}4}}& {\upsigma }_{{\text{u}}_{\text{ADWG}2},{\text{u}}_{\text{ADWG}4}}& {\upsigma }_{{\text{u}}_{\text{ADWG}3},{\text{u}}_{\text{ADWG}4}}& {\upsigma }_{{\text{u}}_{\text{ADWG}4}}^{2}\end{array}\right],$$ for example, ${\upsigma }_{{\text{u}}_{\text{ADWG}1}}^{2}$ is the genetic variance of ADWG_1_, ${\upsigma }_{{\text{u}}_{\text{ADWG}2}}^{2}$ is the genetic variance of ADWG_2_ and ${\upsigma }_{{\text{u}}_{\text{ADWG}1},{\text{u}}_{\text{ADWG}2}}$ the genetic covariance between the two environmental levels. The other elements of $\mathbf{T}$ are the remaining pair-wise covariances with the genetic variance on the diagonal. The random residual effects were distributed as $$\left[\begin{array}{c}{\mathbf{e}}_{\mathbf{A}\mathbf{D}\mathbf{W}\mathbf{G}1}\\ {\mathbf{e}}_{\mathbf{A}\mathbf{D}\mathbf{W}\mathbf{G}2}\\ {\mathbf{e}}_{\mathbf{A}\mathbf{D}\mathbf{W}\mathbf{G}3}\\ {\mathbf{e}}_{\mathbf{A}\mathbf{D}\mathbf{W}\mathbf{G}4}\end{array}\right]\sim \text{ N}\left(0, {\varvec{\Sigma}}  \otimes  \mathbf{I}\right)$$, where $${\varvec{\Sigma}}= \left[\begin{array}{cccc}{\upsigma }_{{\text{e}}_{\text{ADWG}1}}^{2}& 0& 0& 0\\ 0& {\upsigma }_{{\text{e}}_{\text{ADWG}2}}^{2}& 0& 0\\ 0& 0& {\upsigma }_{{\text{e}}_{\text{ADWG}3}}^{2}& 0\\ 0& 0& 0& {\upsigma }_{{\text{e}}_{\text{ADWG}4}}^{2}\end{array}\right],$$ ${\upsigma }_{{\text{e}}_{\text{ADWG}(1-4)}}^{2}$ is the residual variance of ADWG_(1-4)_ and $\mathbf{I}$ is an identity matrix. The reverse diagonal of ${\varvec{\Sigma}}$ contained all 0 values because no animals had repeated observations for the same trait in multiple environmental levels. This model was identical to that used for THI environmental levels, the only difference being the distribution of animals across three levels instead of four.

The off-diagonal values were also 0 as the covariances between animal and environmental effects and the residual covariances were assumed to be zero due to no animal was present in multiple environments [35]. The multivariate analysis was performed using MTG2, fitting the same fixed and random effects previously described [34].

### Simulated phenotypes

To determine the impact of shared genetic linkage on the estimates of genetic correlations between environments, we simulated a data set under the null hypothesis of no GxE. The real, imputed genotypes from the population were used, and 1000 replicates of the simulated phenotypes were produced as quantitative traits using genome-wide complex trait analysis (GCTA) [30] by the simple additive genetic model $\mathbf{y}=\mathbf{Z}\mathbf{u}+\mathbf{e}$ [30]. The simulated phenotypes were generated using all 709,768 SNPs as QTL and ${h}^{2}$ was set as the estimated heritability of the real trait (CL presence, first and second pregnancy, respectively) [30]. This heritability was based on the heritability for CL presence from the real data, calculated above. The simulated phenotypes were transformed from a continuous to binary scale based on the distribution of the simulated phenotypes and the proportion of pubertal/non-pubertal or pregnant/non-pregnant in the real phenotype. GCTA does not have a facility to simulate binomial traits. Hence the transformation from continuous to binary, based upon the distribution/proportion of the real traits, was necessary. For example, the proportion for simulated CL presence was 43% reflecting the percentage of heifers that were pubertal at the time of trait recording, the simulated phenotypes were distributed to reflect this. These transformed simulated phenotypes were then analysed using the same multi-trait model as the real phenotypes, estimating the genetic correlation between each environmental level 1000 times to generate an empirical null distribution. This distribution was then directly compared to the correlation between levels for the real phenotypes. GxE was deemed to be statistically significant if the correlations for the real phenotypes fell within the 5th percentile of the distribution of simulated correlations (*P* < 0.05). The heritability of the transformed phenotypes aligned closely to the estimated heritability of the real traits (e.g. 0.15(0.02) for first pregnancy).

## Results

### Genetic linkage between environment levels for ADWG

**Fig. 2:**
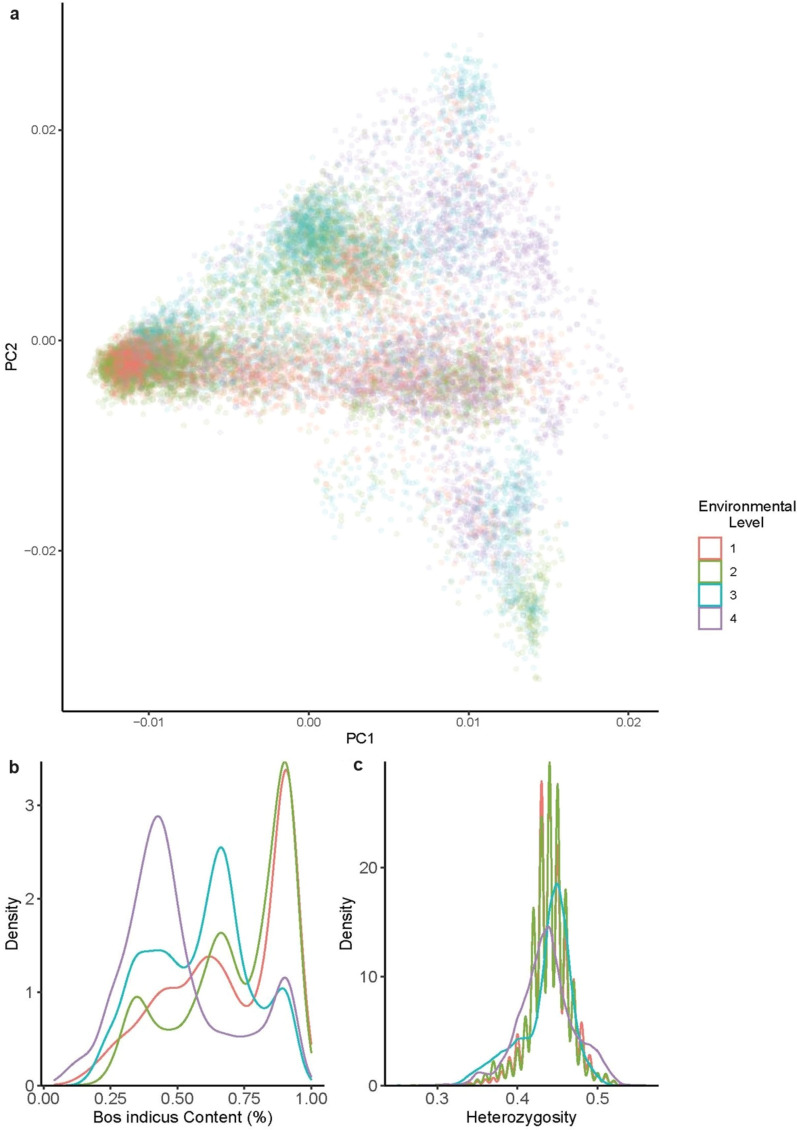
Genetic linkage across average daily weight gain (ADWG) levels within first pregnancy subset. **a** Principal component analysis coloured by environmental level; **b** Density plot showing distribution of *Bos indicus* content within each environmental level for first pregnancy subset; and **c** Density of heterozygosity content for first pregnancy subset

A principal component analysis (PCA) of genetic linkage across the four ADWG environmental levels at first pregnancy (n = 13,549), found principal component 1 to be highly correlated to *B. indicus* content (r^2^ = 0.98) (Fig. 2a). The PCA suggests that while some clustering of genotypes does occur within environment levels, a large degree of overlap is also observed. Fig. 2b confirmed these findings, indicating cattle with higher *B. indicus* content were more concentrated in harsher environmental levels (1 and 2). Although higher heterozygosity was more concentrated in harsher environments, its overall distribution was similar for all four ADWG levels (Fig. 2c).

### Average daily weight gain environmental levels

Both pregnancy and puberty generally increased as nutrition improved with level (Table 1). This suggests that the quartiles were accurately reflecting a change in environmental conditions. Heritability varied considerably when estimated from a univariate analysis within each environmental level, although standard errors on these estimates were large (See Additional file 1: Table S1). The pairwise F_st_ results show the genetic variance within a subpopulation, in this case between the environmental levels, in comparison to the total population. Low F_st_ values suggested limited genetic differentiation between subpopulations, which provided sufficient evidence to examine GxE. However, the lack of clear differentiation does not convey the degree of similarity between environmental levels, especially considering the diversity inherent within the dataset (See Additional file 1: Table S1).

**Table 1:** Information regarding success rates for CL presence, first pregnancy and second pregnancy overall and within environmental levels

Trait^a^	ADWG quartile	n	ADWG (kg/d)^b^	Positive (%)^c^	h^2^ (SE)
CL presence		11,801	0.42	47.21	0.30 (0.02)
	Level 1	3641	0.20	44.96	
	Level 2	2658	0.33	45.74	
	Level 3	2706	0.46	46.32	
	Level 4	2796	0.68	52.08	
First pregnancy		13,459	0.42	71.22	0.15 (0.02)
	Level 1	2991	0.20	64.03	
	Level 2	3934	0.33	75.67	
	Level 3	3058	0.46	75.83	
	Level 4	3152	0.68	79.98	
Second pregnancy		10,044	0	63.99	0.08 (0.02)
	Level 1	1882	– 0.14	61.90	
	Level 2	2717	– 0.03	58.08	
	Level 3	3438	0.04	59.80	
	Level 4	1287	0.18	81.28	

^a^*Corpus luteum* (CL) presence, assessed by ultrasound at about 600 days of age, first pregnancy = first pregnancy test conducted at ~2.5 years of age, second pregnancy = second pregnancy test conducted at ~3.5 years of age.

^b^ADWG = average daily weight gain in kg/d for the entire cohort and within each environmental level.

^c^Positive (%) = This number reflects the percentage of animals that were pubertal at CL presence recording OR were pregnant at first/second pregnancy recording. Values expressed for whole cohort and within each environmental level**.**

**Fig. 3:**
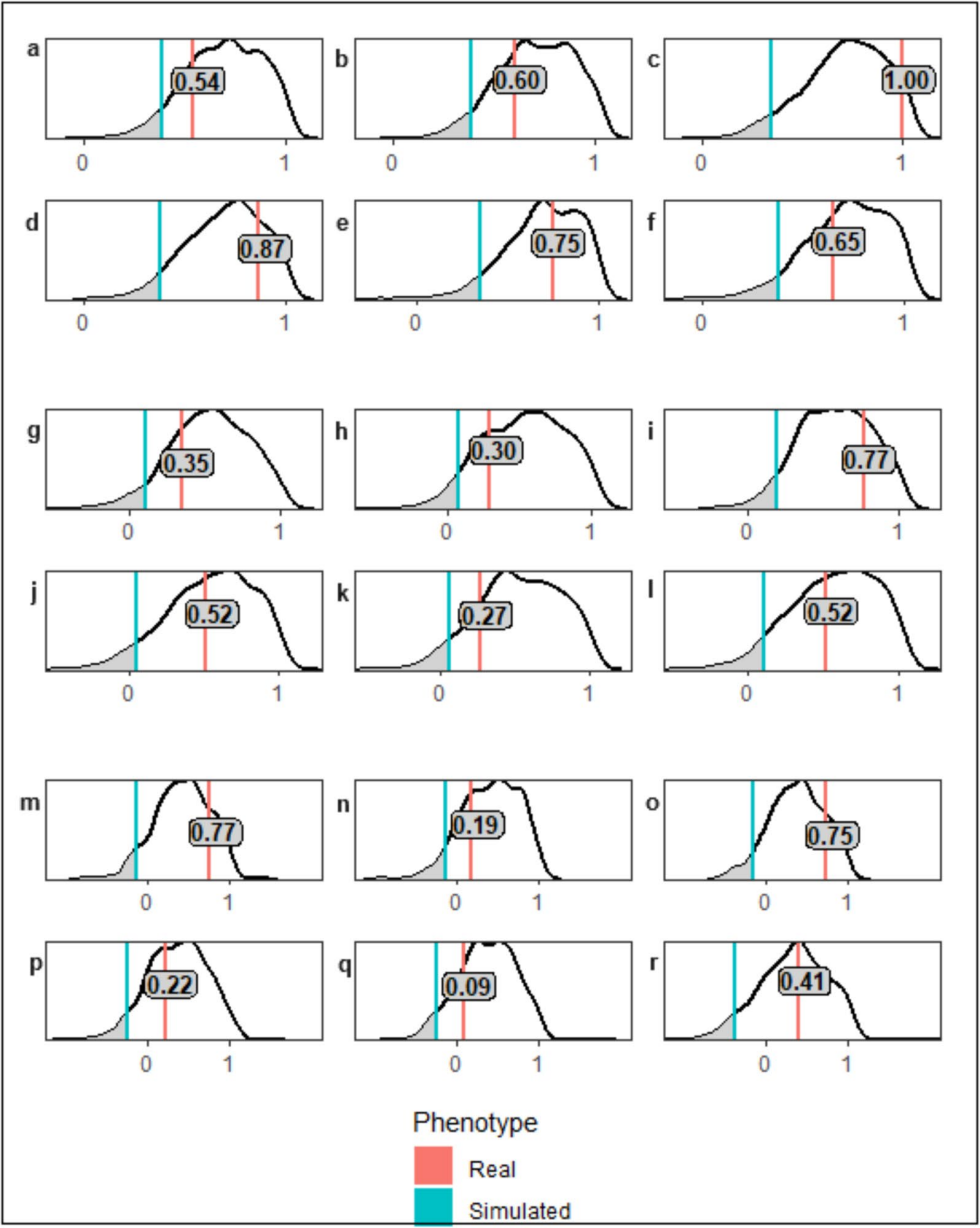
Genetic correlations between environmental levels for simulated and real phenotypes for average daily weight gain. Red = genomic correlation based on real phenotypes, blue = 5th Percentile values for simulation correlation distribution (P < 0.05). Genetic correlation for real phenotypes in bold on red line. **a**–**f** CL presence correlations for ADWG environmental levels **a** 1v2, **b** 1v3,** c** 2v3, **d** 1v4, **e** 2v4, **f** 3v4. **g**–**l** First Pregnancy correlations for ADWG environmental levels **g** 1v2, **h** 1v3, **i** 2v3, **j** 1v4, **k** 2v4, **l** 3v4. **m**–**r** Second Pregnancy correlations for ADWG environmental levels **m** 1v2, **n** 1v3, **o** 2v3, **p** 1v4, **q** 2v4, **r** 3v4

Estimated genetic correlations between the ADWG environmental levels for the real and simulated phenotypes were determined (Fig. 3, Additional file 1: Table S1). Genetic correlations of < 0.8, when taken in isolation, are typically indicative of GxE [17]. However, this analysis compared the correlation between the real phenotypes in each environment to the null distribution generated from the 1000 replicates of the simulated correlations. It was determined that GxE was only observed when the correlation for the real phenotypes was below the 0.05 significance level of the null distribution. Based on this criterion, no statistically significant observations of GxE were made for fertility traits across the ADWG environmental levels (Fig. 3).

### Temperature humidity index environmental levels

The percentage of pubertal heifers declined in the reverse to the expected direction with heifers in the ‘harshest’ level indicating the highest percentage pubertal (Table 2). This trend may have been associated with *B. indicus* content, which was generally higher in harsher environments. However, any confounding impact of this trend was minimised by accounting for *B. indicus* content in the multi-trait model. This trend was less pronounced for first and second pregnancy where the response to the THI environmental gradient was ill-defined in either direction (particularly for environmental levels 1 and 2, more defined comparing 2 and 3) (Table 2). The heritability of each trait was calculated within each environmental level subpopulation (See Additional file 2: Table S2).

**Table 2:** Descriptive statistics for CL presence, first pregnancy and second pregnancy overall and within environmental levels

Trait^a^		n	n days THI > 65^b^	Positive (%)^c^	h^2^ (SE)
CL presence		21,037	79.6	43.37	0.27 (0.01)
	L1	6740	115.0	48.39	
	L2	5483	86.8	44.12	
	L3	8814	38.7	38.67	
First pregnancy		13,549	115.2	73.59	0.15 (0.02)
	L1	4612	120.00	77.21	
	L2	4736	119.94	78.45	
	L3	4201	105.70	64.17	
Second pregnancy		10,044	114.2	63.99	0.18 (0.02)
	L1	2475	120.00	56.42	
	L2	2519	119.51	67.89	
	L3	5057	102.41	65.89	

^a^CL presence = ovarian ultrasound scan and 600 days of age, First pregnancy=first pregnancy test conducted at ~2.5 years of age, Second pregnancy=second pregnancy test conducted at ~3.5 years of age.

^b^n days THI >65 = number of days where daily peak temperature humidity index exceeded 65 in the 120 days prior to trait recording for CL presence and surrounding conception for first and second pregnancy.

^c^Positive (%) = This number is the percentage of animals that were pubertal at CL presence recording OR were pregnant at first/second pregnancy recording as a whole and also within each environmental level

The genetic linkage between environmental levels was initially examined via a pairwise F_st_ analysis. These results indicated a similar pattern observed across the ADWG levels, where relative genetic distance was higher between environments at opposing ends of the environmental spectrum (See Additional file 2: Table S2). However, the pairwise Fst estimates were still universally low, indicating that subpopulations were not significantly differentiated from each other. A principal component analysis suggested that there was substantial genetic overlap between environmental levels (See Additional file 3: Figure S1).

**Fig. 4:**
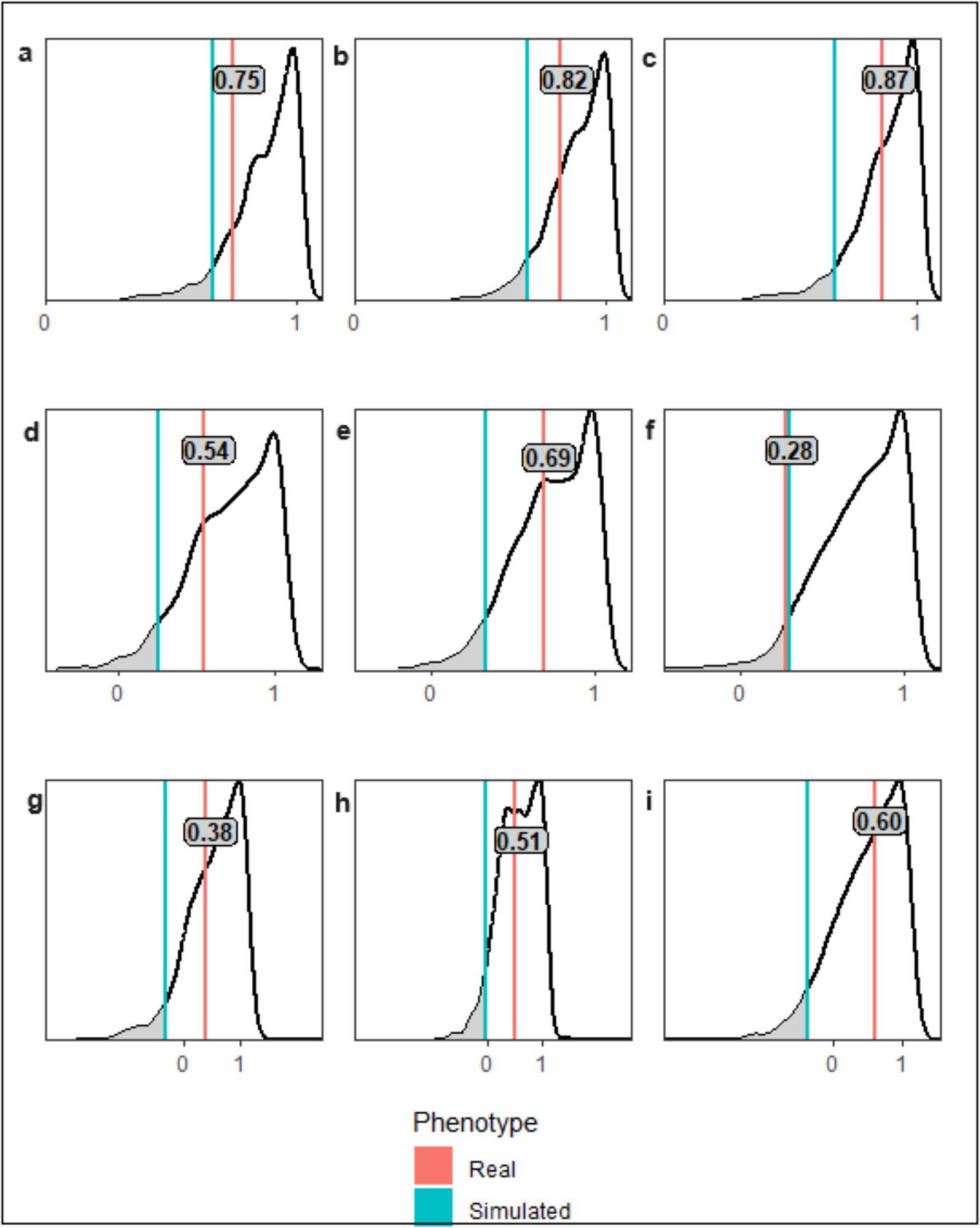
Genetic correlations between environmental levels for simulated and real phenotypes for temperature humidity index. Red = genomic correlation based on real phenotypes, blue = 5th Percentile values for simulation correlation distribution (P < 0.05). Genetic correlation for real phenotypes in bold on red line. **a**–**c** CL presence correlations for THI environmental levels **a** 1v2, **b** 1v3, **c** 2v3. **d**–**f** First Pregnancy correlations for THI environmental levels **d** 1v2, **e** 1v3, **f** 2v3. **g**, **h** Second Pregnancy correlations for THI environmental levels **g** 1v2, **h** 1v3, **i** 2v3

In contrast to ADWG levels, genetic correlations between THI levels for CL presence were found to be high, almost universally >0.8, the only exception being the correlation between levels 1 and 2, an estimate that was within the standard error of the defined threshold of 0.8 (Fig. 4, Additional file 2: Table S2). When the genetic correlations were compared to the null distribution of correlations from the simulated phenotypes, the only statistically significant result (*P* < 0.05) was for first pregnancy between levels 2 and 3 (Fig. 4). Notwithstanding this marginal example, no other significant (*P* < 0.05) evidence of GxE was found for fertility traits between THI environmental levels.

## Discussion

This study found no significant evidence of GxE for fertility traits across ADWG environments and only limited evidence across THI environmental levels. The environmental descriptors used to define the multi-trait model were designed to measure the chief challenges faced by breeding females in northern Australia, namely nutrition deficiencies and exposure to high temperatures [5, 36, 37]. Examining the genetic correlations for the real phenotypes in isolation may suggest some GxE (r < 0.8), however comparison to an empirical distribution derived from 1000 simulated phenotypes found that these indications are more likely an artefact of the genetic structure of the dataset, specifically lack of genetic linkage across environment levels. Therefore, our results serve to illustrate the difficulty of assessing GxE in such datasets. Prior studies examining GxE have been limited to smaller, intensively managed herds where genetic linkage between environmental levels can be assured [3, 11]. Other studies in industry datasets have not focussed on fertility traits [20, 38]. The absence of significant GxE suggests that selection for the examined traits can be accurately carried out across a wide variety of environments for this population.

### Heritability of fertility traits

The heritability for all three fertility traits was re-estimated, as trait definitions differed slightly from those calculated by [15]. Our results for heritability were similar to those reported by Corbet et al. [22] of 0.18-0.32, found in sub-populations of different breeds, from the closely aligned trait of reproductive maturity score. The estimates made by Hayes et al.[15] for *corpus luteum* score (0.25) and *corpus luteum* rate (0.22) also supported our results. Estimated genomic heritability of pregnancy traits were comparable to those reported by Corbet et al. [22] who investigated pregnancy rate in maiden Brahman heifers (0.15 (0.05)) and first lactation Brahman cows (0.17 (0.10)). Guarini et al. [39] found heifer heritability of heifer rebreed rate, comparable to second pregnancy, to be 0.15 (0.02). Our estimates for second pregnancy were lower than those for second pregnancy rate in Brahman cows (0.35) found by [40]. Our results, especially for CL presence, demonstrate the value in identifying reproductive traits which can be measured early in life and are moderately to highly heritable. Fertility traits can be difficult and expensive to measure, and low heritability generally undermines, or at least slows, the benefits of selecting for fertility. Traits like CL presence with moderate to high heritability mean that selection decisions can be made early and with confidence.

### Simulated phenotypes

The dataset used was typical of northern Australia, with a population with no pedigree information, unknown genetic linkage, and a multibreed heritage [15]. Recorded animals were bred under commercial conditions representative of the environmental and economic realities of extensive grazing systems. These realities are seldom reflected in research herds and using an unstructured dataset from this production system poses significant challenges. The greatest challenge is that traditional pedigree or parentage-based methods to ensure sufficient genetic linkage between environmental categories are unavailable. This has limited the utility of industry datasets for assessing GxE in the past and was overcome in this study by using SNP array genotypes. The benefits of exploiting SNP array genotypes in relation to GxE studies have previously been confirmed by Mulder [7]. When genotypes are used, the key requirement for the accurate estimation of GxE is that chromosome segments or SNP alleles are replicated across environments. We used simulations to assess the impact of the degree of replication of chromosome segments/SNP alleles in the data set by using the actual genotypes from the data set to estimate genetic correlations between environmental levels. The risk of over-estimating GxE has generally led to the conclusion that GxE can only be investigated within tightly controlled research herds where the family structure is known [10, 11, 41, 42]. The use of simulated phenotypes has been demonstrated as viable and our study utilised these simulations to create context around the multi-trait models in an industry dataset [43–45]. The use of the simulated phenotypes to create an empirical distribution of correlations created a population specific threshold for GxE, thus helping to distinguish true GxE from background genetic noise. The use of genomic data instead of pedigree information made this examination of GxE feasible. However, whether this calibre of information will be available in commercial herds outside of a project like Northern Genomics remains to be seen.

### Genotype x environment interactions

The results of this study found limited statistically significant evidence of GxE for the three female fertility traits analysed. Limited supporting evidence for this result exists in the literature due to the relative novelty of our dataset and the traits examined. Many of the research populations previously assessed for GxE interaction are not representative of the commercial beef industry either in scale or in breed composition. The focus has largely been on temperate, *B. taurus* breeds in relatively moderate environments [11, 46–48]. These environments do not represent the stress faced by animals in northern Australia which ranges from dry arid conditions to very high rain fall, high humidity environments. Some relevant studies examining weight traits for GxE in tropically adapted populations have been carried out; in these cases, sire re-ranking was observed and genetic correlations were found to decrease, indicating GxE, as the environments diverged [38, 49]. However, thorough examinations of fertility traits for GxE in similar populations, are less available. Santana et al. [35] detected some indications of GxE for male fertility traits in tropically adapted cattle under tropical conditions, but their study did not provide a comparison for female fertility traits.

The lack of evidence of GxE across ADWG of THI environmental levels indicates that, for fertility traits at least, genomic evaluation and selection do not need to be split according to environment [17]. It has been previously noted that the greatest potential for re-ranking occurs in moving from temperate or subtropical environments to less favourable conditions [6, 46]. If re-ranking was occurring for fertility traits, animals of the highest genetic merit for their respective environments would potentially not be identified and the results of targeted selection for fertility would be reduced. Our results suggest, however, that this is not the case. Accurate selection to improve fertility is beneficial to the profitability of the northern beef industry [50]. The results of this study indicate that fertility traits may be accurately selected for in any environment without the need to use environment specific breeding values [17].

While the lack of GxE for fertility traits simplifies selection, it does not remove the requirement for cattle that are properly adapted to their environmental conditions. Heat stress has a well-proven effect on fertility outcomes, including the suppression of follicle stimulating hormone secretion from the pituitary gland, which slows or stops the reproductive cycle [51–54]. The effect of heat stress on fertility is most readily apparent in dairy breeds [19, 55, 56] and *Bos taurus* beef cattle in tropical conditions [51, 57]. However, the effect of heat stress on both reproductive outcome and oocyte survival was found to be less pronounced in *B. indicus* cows [51, 57]. Clearly then, the use of *B. indicus* cattle with some level of adaptation is still strongly recommended in the context of northern Australia, despite the lack of evidence for GxE in fertility traits in this study.

### Potential drawbacks and future work

The unique nature of our study, applying a multivariate GxE analysis to a diverse industry dataset, provided an excellent opportunity to leverage a commercial beef dataset to deliver relevant and applicable results directly back to the industry. However, the strength of the data as a reference dataset for commercial herds also created some significant challenges. The first key challenge was the lack of precision and consistency in the timings of trait recordings, potentially affecting the accuracy of the measurements of ADWG which were the cornerstone of the GxE analysis. The raw mean of ADWG data, as adopted in this study, acted as a proxy for environmental ‘harshness’ where the exact reasons for reduced weight gain, such as disease or drought, were unknown. Animals had, at most, three weight records, thus the dataset was unable to capture weight gain at specific points of peak nutritional requirement, such as prior to joining. Additionally, the lack of weight records on animals prior to CL presence undermined the examination of GxE across ADWG levels for what was the most heritable trait with the highest number of records. Future work with this or related datasets could potentially collect and leverage weight records directly from project collaborators to help fill this gap.

Lack of precision was also an issue for the THI environmental descriptors. Attempts to model acute heat stress events (e.g., THI > 79) in the same dataset using linear modelling were previously unsuccessful [4]. The heat load descriptors analysed here were developed from an interpolated dataset rather than direct measurements. Identifying critical time periods of acute heat stress has previously been done successfully in dairy cattle by Shi et al. [58] and in pigs by Freitas et al. [59]. Both of these studies sought to investigate GxE for fertility traits and potentially identify environmentally robust animals, however these studies were conducted on intensively managed livestock where there was virtually unlimited access to the animals for trait recording [58, 59]. In the case of Shi et al. [58], measurements of temperature and humidity information were made hourly and thus a precise comparison could be made between reproductive traits and environmental conditions. It was not possible to capture that level of detail in our study where, for example, conception or calving dates were based entirely on back or forward-dating from the pregnancy test date with the estimated foetal age [4, 21]. The heat load descriptors we adopted were not designed to capture this level of detail, rather, they were intended to function as proxies for the relative level of environmental harshness.

In spite of these potential drawbacks, our study provided a thorough examination of GxE for fertility traits in relation to the two most pressing challenges for cattle in northern Australia, high heat stress and nutritional availability. Additional precision, as is typically desired for GxE studies, simply was not achievable. The greatest strength of this dataset remains its representation of the diverse breeds, management systems, environmental challenges and reproductive performance observed in northern Australia. Future efforts will explore utilizing a random regression reaction norm to study the presence of GxE in this population.

## Conclusions

Limited significant indications of GxE for fertility traits in this population were found using a multi-trait approach for estimating GxE. Initial indications based only on genetic correlations estimated from real phenotypes proved to be biased when compared to an empirical null distribution of correlations derived from simulated phenotypes. Further work is required to probe the presence of GxE for fertility and production traits given the genetic structures present within this industry dataset. If substantiated, the absence of GxE across ADWG and THI environmental levels holds particular significance for genetic selection in northern Australia. It would allow for accurate and direct selection for fertility traits, regardless of environment.

## Supplementary Information

Additional file 1: Table S1. Heritability (on diagonal), genetic correlation of REAL phenotypes (above diagonal, genetic correlation of SIMULATED phenotypes (below diagonal) with standard error in brackets for CL presence, first pregnancy and second pregnancy within environmental levels defined by ADWG. The results from earlier figures displayed in table format.

Additional file 2: Table S2. Heritability (on diagonal), genetic correlation of REAL phenotypes (above diagonal, genetic correlation of SIMULATED phenotypes (below diagonal) with standard error in brackets for CL presence, First Pregnancy and Second Pregnancy of heifers within environmental levels defined by THI. The results from earlier figures displayed in table format.

Additional file 3: Figure S1. Genetic linkage across Temperature Humidity Index (THI) levels within CL presence subset. (a) Principal component analysis coloured by environmental level; (b) Density plot showing distribution of *Bos indicus* content within each environmental level for CL presence subset; and (c) Density of heterozygosity content for CL presence subset.

## Data Availability

The data and relevant research materials including scripts can be provided upon request.
