# Supplementary material for: Investigating genotype by environment interaction for beef cattle fertility traits in commercial herds in northern Australia with multi-trait analysis
Source: Genet Sel Evol. 2024 Oct 31;56:70. doi: 10.1186/s12711-024-00936-0 (PMC11526658; doi:10.1186/s12711-024-00936-0)
Supplement: Supplementary file 1 — Additional file 1: Table S1. Heritability (on diagonal), genetic correlation of REAL phenotypes (above diagonal, genetic correlation of SIMULATED phenotypes (below diagonal) with standard error in brackets for CL presence, first pregnancy and second pregnancy within environmental levels defined by ADWG. The results from earlier figures displayed in table format. [file 12711_2024_936_MOESM1_ESM.docx]

| **Trait^a^** | **ADG Lvl^b^** | **1** | **2** | **3** | **4** |
| --- | --- | --- | --- | --- | --- |
| CL presence | 1 | 0.56(0.08) | 0.54(0.14) | 0.60(0.14) | 0.87(0.14) |
|  | 2 | 0.002 | 0.38(0.08) | 1.00(0.15) | 0.75(0.16) |
|  | 3 | 0.014 | 0.011 | 0.32(0.08) | 0.65(0.16) |
|  | 4 | 0.020 | 0.016 | 0.003 | 0.33(0.08) |
|  |  |  |  |  |  |
| First Pregnancy | 1 | 0.58(0.28) | 0.35(0.17) | 0.30(0.21) | 0.52(0.21) |
|  | 2 | 0.001 | 0.31(0.25) | 0.77(0.20) | 0.27(0.24) |
|  | 3 | 0.012 | 0.012 | 0.25(0.07) | 0.52(0.26) |
|  | 4 | 0.022 | 0.023 | 0.004 | 0.25(0.07) |
|  |  |  |  |  |  |
| Second Pregnancy | 1 | 0.33(0.13) | 0.77(0.35) | 0.19(0.47) | 0.22(0.39) |
|  | 2 | 0.001 | 0.46(0.11) | 0.75(0.42) | 0.09(0.33) |
|  | 3 | 0.003 | 0.006 | 0.42(0.19) | 0.41(0.48) |
|  | 4 | 0.001 | 0.013 | 0.004 | 0.63(0.21) |
| ^a^CL presence = ovarian ultrasound scan and 600 days of age, First pregnancy = first pregnancy test conducted at ~2.5 years of age, Second pregnancy = second pregnancy test conducted at ~3.5 years.  ^b^ADWG=average daily weight gain. | | | | | |

**Heritability within, genetic correlations and Fst results between, ADWG environmental levels.**
