# Supplementary figures and images for: Investigating genotype by environment interaction for beef cattle fertility traits in commercial herds in northern Australia with multi-trait analysis
Source: Genet Sel Evol. 2024 Oct 31;56:70. doi: 10.1186/s12711-024-00936-0 (PMC11526658; doi:10.1186/s12711-024-00936-0)

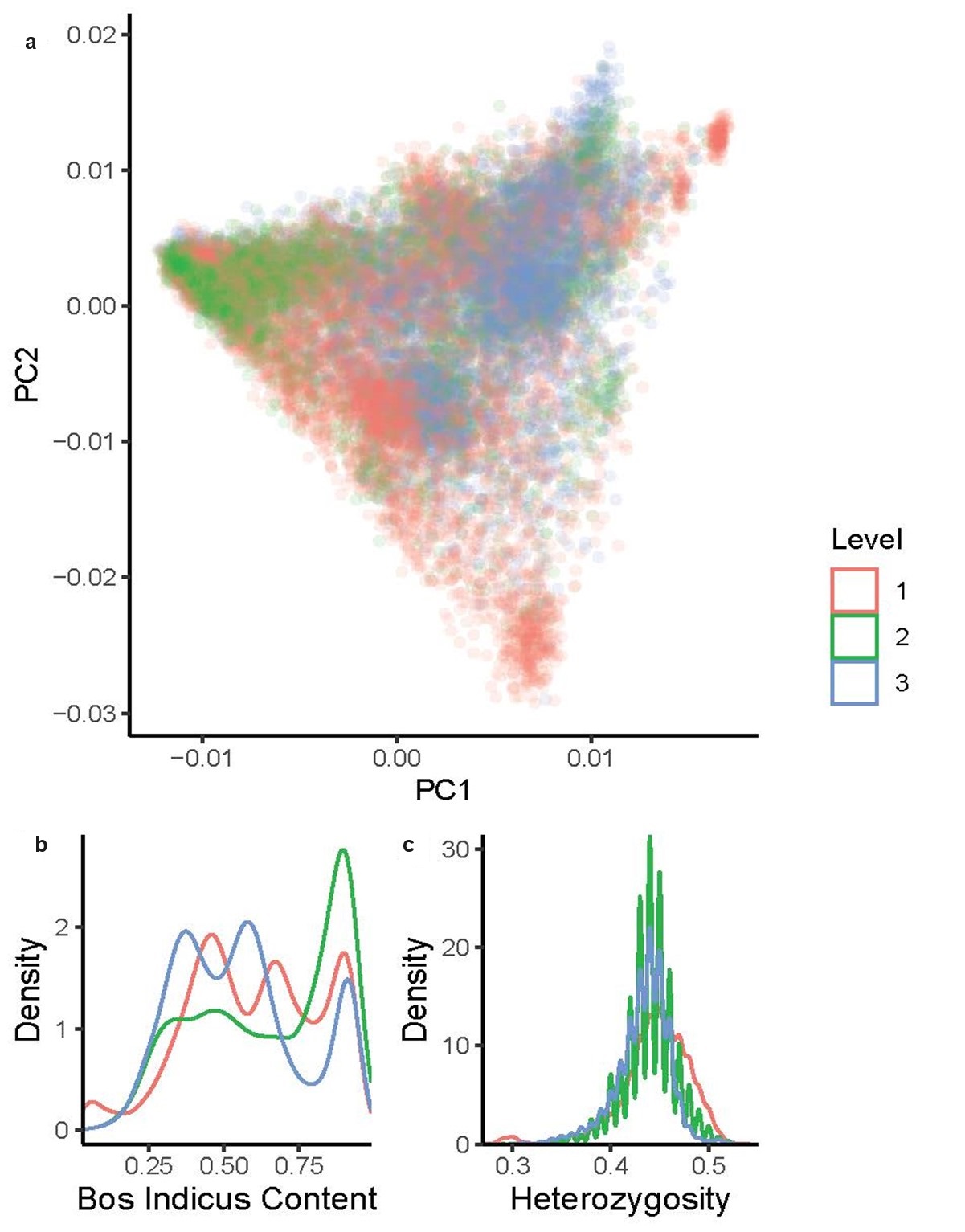

Supplement: Supplementary file 3 — Additional file 3: Figure S1. Genetic linkage across Temperature Humidity Index (THI) levels within CL presence subset. (a) Principal component analysis coloured by environmental level; (b) Density plot showing distribution of Bos indicus content within each environmental level for CL presence subset; and (c) Density of heterozygosity content for CL presence subset. [file 12711_2024_936_MOESM3_ESM.jpg]
